# Lights and shadows on local recurrence after renal surgery: when, why and how to manage

**DOI:** 10.3389/fruro.2024.1419418

**Published:** 2024-05-22

**Authors:** Luca Di Gianfrancesco, Alessandro Crestani, Antonio Amodeo, Paolo Corsi, Davide De Marchi, Eugenio Miglioranza, Giuliana Lista, Ferdinando Daniele Vitelli, Francesca Simonetti, Gian Maria Busetto, Ugo Giovanni Falagario, Martina Maggi, Filippo Marino, Giannicola Genovese, Roberto Falabella, Angelo Porreca

**Affiliations:** ^1^ Department of Oncological Urology, Veneto Institute of Oncology (IOV), Padua, Italy; ^2^ Department of Urology, Azienda Sanitaria Universitaria Friuli Centrale, Udine, Italy; ^3^ Department of Medical and Surgical Sciences, Ospedale Riuniti, University of Foggia, Foggia, Italy; ^4^ Department of Urology, Sapienza University, Rome, Italy; ^5^ Department of Urology, Fondazione Policlinico Universitario Agostino Gemelli IRCCS, University of Sacred Heart, Rome, Italy; ^6^ Department of Genitourinary Medical Oncology and Genomic Medicine, University of Texas MD Anderson Cancer Center, Houston, TX, United States; ^7^ Department of Urology, Azienda Ospedaliera Regionale San Carlo, Potenza, Italy

**Keywords:** local recurrence, partial nephrectomy, positive surgical margin, prognostic factor, review, renal cell carcinoma

## Abstract

**Introduction:**

This review aims to analyze the existing literature on local recurrence (LR) in patients undergoing partial nephrectomy (PN) for renal cell carcinoma, identifying relative risk factors, and exploring optimal clinical management strategies.

**Methods:**

A comprehensive literature search was conducted across bibliographic databases, primarily focusing on LR rates. Secondary outcomes included evaluation of positive surgical margins (PSM), nephrometry scores, pathological stage (T and grading), perioperative outcomes, time-to-LR, overall survival, and cancer-specific survival.

**Results:**

Due to the heterogeneity, a narrative synthesis was performed. LR rates after PN varied in the literature; with PSM emerging as a significant risk factor. Other LR risk factors included pathological stage, nephrometry scores, and histological variants. However, evidence regarding optimal LR management in the absence of precise indications was lacking.

**Conclusion:**

LR represents a significant clinical challenge; requiring multidisciplinary assessment and shared decision-making with patients. Given well-established risk factors, clinicians must tailor management strategies to optimize patient outcomes.

## 1 Introduction

International guidelines suggest partial nephrectomy (PN) as the current standard-of-care for cT1a and most cT1b renal cell carcinoma (RCC) cases (1). However, conservative surgery may be negatively compromised by the risk of positive surgical margins (PSM), which is one of the most significant factors contributing to local recurrence (LR) (2). LR risk is notably influenced by PSM after PN, particularly in T2 RCC cases, as indicated by past investigations (16% vs 3% in patients with negative surgical margins (NSM) (3, 4). While various approaches to managing PSM have been suggested, there is no widely accepted guideline to determine the optimal modality and timing of treatment (5).

The main goal of nephron-sparing surgery (NSS) is to achieve optimal oncological control while preserving overall renal function and minimizing perioperative complications. However, a potential limitation of NSS is the occurrence of PSM in 0.1–10.7% of cases (6). Although, international guidelines recommend a strict follow-up after evidence of PSM, consensus on a specific strategy is lacking, and the association between PSM and LR remains under debate (1). While several studies have shown no correlation between PSM and a higher risk of metastases or decreased cancer-specific survival (CSS) (3), large retrospective studies have identified PSM as an independent predictor of recurrence (7, 8). Moreover, the absence of PSM, as seen in Trifecta achievement (negative surgical margins, warm ischemia time <25 minutes, and no complications) (9), has been reported to play a role in predicting long-term outcomes after robot-assisted partial nephrectomy (RAPN) (10).

In this study, we reviewed the literature to identify risk factors for LR in patients undergoing PN for RCC and to provide an overview of the current evidence.

## 2 Materials and methods

### 2.1 Data acquisition and search strategy

**Figure 1:**
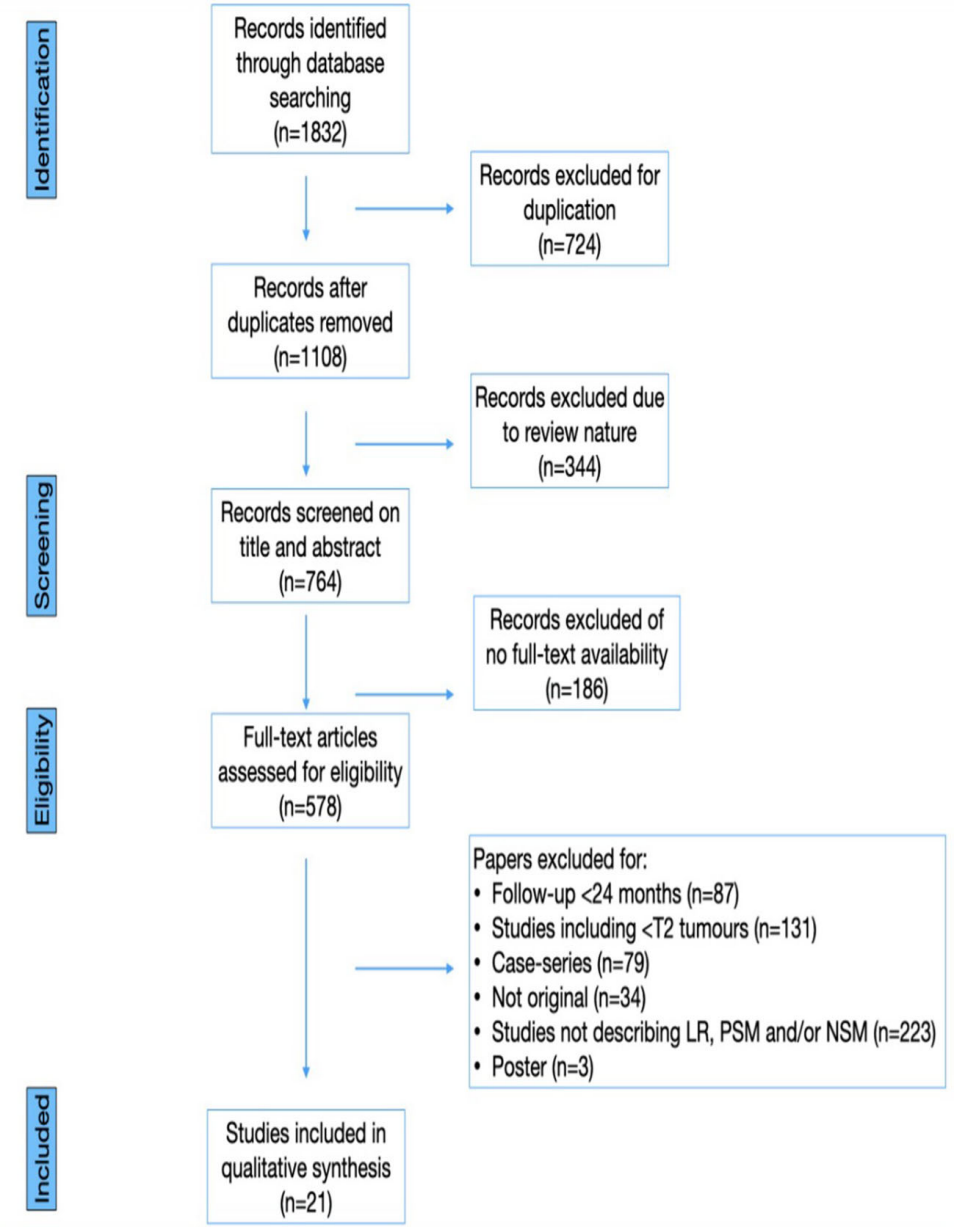
PRISMA flow-chart.

A comprehensive literature search was conducted, encompassing papers published until March 1st,2023. The search was performed on Pubmed, Web of Science, and Clinicaltrials.gov, using the following key words (title and abstract): “renal carcinoma” or “kidney cancer” or “local recurrence” or “positive surgical margin” or “negative surgical margin” or “robot-assisted surgery” or “partial nephrectomy” or “robot-assisted partial nephrectomy”. Additionally, a supplementary search of grey literature was performed using Google Scholar. References of significant articles were also manually analyzed to identify other relevant citations. Case reports, letters to the editor, editorials, congress abstracts and studies involving pediatric patients were excluded. All abstracts and full-text articles were independently reviewed, adhering to pre-defined inclusion and exclusion criteria to retrieve relevant articles. This systematic review was conducted according to the Cochrane Handbook (10) and the Preferred Reporting Items for Systematic Reviews and Meta-analysis (PRISMA) criteria (11). **Figure 1** presents a PRISMA flowchart detailing the article identification process.

### 2.2 Types of studies and participants

Retrospective and prospective cohort studies investigating LR and surgical margins in patients undergoing PN for RCC were deemed eligible for inclusion, without restrictions based on histology. Additionally, patients who underwent salvage radical surgery following the detection of PSM were included. In instances where multiple papers reported the same outcome using identical study cohort, data from the most recent paper were utilized. We extracted data from all pertinent publications and conducted an analysis to determine whether different outcomes from similar study cohorts were reported.

### 2.3 Types of outcome measures included

The main focus of this review was to measure LR, quantified either as the absolute number of events or as a percentage. LR was determined based on radiological imaging and the diagnosis of recurrence at the tumor bed or near the site of the original tumor in the ipsilateral kidney. Additionally, we assessed the surgical margin of the PN specimen, as evaluated by a pathologist on final pathology. PSM was defined as the presence of tumor cells in contact with the stained area of the specimen during histological evaluation, while NMS referred to the absence of contact between tumor cells and the stained area of the specimen.

### 2.4 Data synthesis

We collected and recorded data, extracting baseline study characteristics and presenting them using descriptive statistics. Initially, the review aimed to incorporate a meta-analysis; however, due to the substantial heterogeneity among the studies, conducting a meta-analysis was not feasible. Therefore, we opted for a narrative synthesis instead. We analyzed various factors separately, including the rates of LR, PSM, nephrometry scores, pathological stage, preoperative outcomes, time-to-LR, overall survival (OS), and CSS.

## 3 Results

### 3.1 Definitions

The literature presents various definitions of PSM and LR, as outlined below.The definition of PSM ranges from instances where no clear definition is provided (12–14) to description such as “malignant cells being present at the inked parenchymal surgical margin of resection on the final pathology assessment” (15), “a large number of residual tumor cells at the surgical margin or incision of satellite tumor nodules around the large tumor” (16), “tumor cell in contact with Chinese ink” (17), “presence of neoplastic cells directly in contact with the inked surface of the specimen” (18), “presence of malignant cells at the surgical margin” (19), and “extension of the tumor to the surface of the specimen in permanent pathology” (20). Similarly, the definition of LR varies from instances where no clear definition is provided (14, 19) to definitions such as “tumor recurrence at the site of the previous PN” (13), “tumor bed recurrence” (15), “*in situ* recurrence” (16), “tumor mass in the ipsilateral kidney over the resection bed of the same histological type of the original tumor” (17), “recurrence at the enucleation site was considered true LR” (18), “new detection of the tumor mass in the same surgery site based on radiographic evidence on chest X-ray, CT scan, MRI, or bone scan with or without pathologic confirmation” (20).

### 3.2 Types of outcome measures included

The literature search yielded a total of 1832 papers, comprising 1646 full-text papers, 344 reviews, and 724 duplicates. After removing duplicates, 578 papers remained for screening. Subsequently, 1254 articles were excluded, and the titles and abstracts of the remaining 578 papers were screened for eligibility. Studies were considered eligible if they included patients who underwent PN for RCC with LR, had either a prospective, randomized clinical trial (RCT) or retrospective design, maintained a mean follow-up of at least 24 months, and reported oncologic outcomes for a minimum of two years. After fulfilling all the inclusion criteria, 21 studies were identified (13–33), and other 15 studies were excluded primarily due to short follow-up durations. **Table 1** summarizes the characteristics of the included studies.

**Table 1:** Characteristics of the included studies.

		Study type	Type of procedure	Tumor size, median (cm)	Follow-up, median (m)	Procedures	PSM (%)	LR (%)	Distant metastases (%)	CFS	OS	CSS
**1**	**Furukawa et al. (24)**	Prospective multicenter	RAPN	2.7	60	103	0	0	4.9	92.8	97.9	100
**2**	**Beauval et al. (21)**	Prospective multicenter	RAPN	3	64.4	110	10	2.7	6.4	86.4	94.9	96.8
**3**	**Wu et al. (14)**	RCT	LPN	3.0	24	15	0	0 in both PSM and NSM	NA	NA	NA	NA
**4**	**Bertolo et al. (28)**	Prospective maintained database	RAPN	3.24	70.8	278	4.3	3.61	3.2	NA	89.9	98.2
**5**	**Carbonara et al. (26)**	RC multicenter	RAPN	3	88	85	8.2	0	2.3	91.7	91.7	97.7
**6**	**Khalifeh et al. (22)**	RC multicenter	RAPN	2.9	63.6	943	2.2	1	0.4	96	88	99.2
**7**	**Minervini et al. (18)**	RC	RAPN	3.0	61	121	2.5	0 in both PSM and NSM	NA	NA	NA	NA
**8**	**Koukourikis et al. (25)**	RC	RAPN	4.2	58	155	10.5	2.1	2.8	93.6	96.7	NA
**9**	**Vartolomei et al. (29)**	RC	RAPN	3	59	90	2.2	2.2	5.5	97.5	95.1	97.5
**10**	**Andrade et al. (33)**	RC	RAPN	2.6	61.9	110	1.7	0	1.7	97.8	91.1	97.8
**11**	**Oh et al. (15)**	RC	OPN and RAPN	2.3/2.2	48.3	702	1.6	0 in PSM, 0.3 in NSM	NA	NA	NA	NA
**12**	**Li et al. (16)**	RC	OPN and LPN	NA	56	600	3.3	0 in PSM, 0.2 in NSM	NA	NA	NA	NA
**13**	**Marchiñena et al. (17)**	RC	OPN, LPN and RAPN	2.9	24	314	7	9.1 in PSM, 1.4 in NSM	NA	NA	NA	NA
**14**	**Chang et al. (31)**	RC	RAPN vs LPN vs OPN	2.8/2.7/2.5	60/60/64	380/206/722	2.5/4.1/1.6	1.5/2.5/1.6	4.9/2.5/1.6	98.4/99.2/98.4	98.4/99.1/98.4	90.2/86.9/88.5
**15**	**Audigé et al. (23)**	RC	RAPN vs OPN	2.96/3.18	85/162	162/167	1/5.9	1.8/6.5	1.8/4.8	5-year approx 85% in both groups	5-year approx 90% in both groups	NA
**16**	**Ali Abdel Raheem et al. (30)**	RC	RAPN vs OPN	2.8/2.5	59/53	52/37	9.6/8.1	2.3/5.9	2.3/2.9	94/95.8	95.5/93.8	100/93.8
**17**	**Wang et al. (32)**	RC	RAPN vs OPN	3.8/3.6	49/52	164/159	5	3.7/1.8	1.2/1.9	95.1/92.7	NA	98.7/97.6
**18**	**Radfar et al. (20)**	RC	OPN and LPN	NA	32.3/32.1	122	34.4	11.9 in PSM, 0 in NSM	NA	NA	NA	NA
**19**	**Kızılay et al. (27)**	RC	RAPN vs LPN	2.48/2.79	58.1/64.8	71/71	2.8/4.2	9.9/14.1	2.9/4.2	97.1/95.8	82.6/84.8	90.1/85.9
**20**	**Çinar et al. (19)**	RC	OPN and LPN	3.53/3.01	28.9	215	7.6	6.3 in PSM, 1.5 in NSM	NA	NA	NA	NA
**21**	**Kang et al. (13)**	RC	NA	2.8/2.5	32.5	1813	1.7	0 in PSM, 0.4 in NSM	NA	NA	NA	NA

RC, retrospective cohort; RCT, randomised clinical trial; RAPN, robot-assisted partial nephrectomy; OPN, open partial nephrectomy; LPN, laparoscopic partial nephrectomy; PSM, positive surgical margin; NSM, negative surgical margin; LR, local recurrence; CFS, cancer-free survival; OS, overall survival; CSS, cancer-specific survival; NA, not available.

All studies had retrospective design, except for those by Furukawa et al. (16), Beauval et al. (21), and Bertolo et al. (28), which reported data from a prospective maintained database. Collectively, the studies included nearly 8000 patients, with mean tumor size ranging from 2.2 cm (15) to 4.2 cm (25). The median overall follow-up was at least 24 months, with 16 studies having a median follow-up longer than 48 months (13, 15, 16, 18, 22–33). Reported rates of PSM ranged from 0% (14, 24) to 34.4% (20). LR was observed in up to 11.9% of open partial nephrectomy (OPN) patients (20), up to 14.1% of laparoscopic partial nephrectomy (LPN) patients (30), and up to 9.9% of RAPN patients (27). Distant metastases were reported in up to 6.4% of patients (21). The 5-year CFS estimates ranged from 86.4% (18) to 98.4% (68), while the 5-year cancer-specific survival (CSS) estimates ranged from 90.1% (27) to 100% (24, 30). Additionally, the 5-year overall survival (OS) estimates ranged from 82.6% (27) to 97.9% (31).

### 3.3 Rate of LR

The rates of LR varied from 0% to 14.1% among patients with PSM (27). Generally, a higher incidence of PSM within the cohort correlated with an increased LR rate. Conversely, only 0% to 1.5% of LR cases were observed in patients with NSM, although this trend was inconsistent. Furthermore, in some cohorts, the LR rate was slightly higher in the NSM group compared to PSM patients (13, 15, 16). In a study utilizing data from a multi-institutional French database, 2.7% of 110 patients developed LR, and 6.4% progressed to metastatic disease after a median follow-up of 64.4 months following RAPN. The cumulative incidence of LR was 3.61% and 4.16%, while the cumulative incidence of metastases was 3.24% and 4.57% at 5 and 7 years, respectively (21). Furthermore, the rate of distant metastases was acceptably low, with a maximum reported rate of 6.4% in a prospective multicenter study (21); this rate also aligns with those reported by historical open and laparoscopic PN series (34).

### 3.4 Surgical margins

The correlation between recurrence rates and surgical margins in the literature lacks consensus. Studies included in evidence synthesis exhibit a wide range of PSM percentages, ranging from 0% to 34.4%. The rate of LR varies from 0% to 9.1% in PSM patients and from 0% to 1.5% in NSM patients. However, many studies are retrospective, and some may also include patients treated during a learning curve, potentially explaining the heterogeneous results regarding PSM rates. PSM could certainly negatively impact oncologic outcomes.

For instance, Kang et al. reported no statistically significant differences in LR rates between PSM and NSM patients (p= 0.492), tumor grade (p= 0.141), or recurrence-free survival (RFS) on Kaplan–Meier analysis (p = 0.566) in a cohort study of 1813 pathology-proven clear cell RCC (ccRCC) (13). Oh et al. observed a smaller width of surgical margins after open or robotic PN in recurrent patients compared to non-recurrent cases (2.26 ± 1.51 mm vs 2.43 ± 2.07 mm; p= 0.218). However, the authors did not specifically analyze LR rates between PSM and NSM patients (15).

Li et al. distinguished between false PSM and true PSM in a cohort of 600 patients treated with PN in China to develop a classification for PSM. They found significantly higher recurrence rates in PSM and true PSM compared to NSM patients (p= 0.0252 and p= 0.0094, respectively). However, the authors did not report higher recurrence rates in false PSM compared to NSM patients (p = 0.3727) (16).

Marchiñena et al. reported PSM (approximately 7%) and LR (approximately 9.1%) rates in a cohort of 314 patients treated with OPN, LPN or RAPN. Moreover, the authors reported a high LR rate (1.4%) in NSM patients in the same cohort (17).

Minervini et al. evaluated oncologic outcomes after robot-assisted tumor enucleation and found PSM in a small percentage of cases (approximately 2.5%), all of which had invasion of pseudocapsules, but no LR events were reported, neither in PSM group nor in NSM group (18).

Çinar et al. reported PSM (approximately 7.6%) and LR (approximately 6.3%) rates in LPN patients for T1a RCC with no significant differences between open and laparoscopic approaches (19).

Wu et al. reported no PSM, LR, or metastasis events in an RCT comparing laparoscopic microwave-assisted enucleation with LPN despite including 21 patients (13.8%) with high-grade tumors after a median follow-up of 24 months (14).

Radfar et al. analyzed 122 patients from a cohort of 750 RCCs treated with PN, focusing on 42 patients with PSM and 80 NSM patients. LR events were observed in 5 patients with PSM, while no events were reported in the NSM group (20).

Khalifeh et al. reported the oncological outcomes of 21 patients with PSM from a cohort of 947 RAPNs performed for RCC. They observed 9 cases of LR and 4 cases of metastases during a median follow-up of 13 months. PSM showed a significant association with recurrence (adjusted HR of 18.4). Additionally, PSM patients exhibited lower rates of RFS and metastasis-free survival (MFS) than those with NSM after 3 years of follow up (47% vs. 98.3%, and 63% vs. 99.5%, respectively) (22).

### 3.5 Pathological stage (T and grading) and histological variants

Marchiñena et al. identified PSM and Fuhrman grade ≥III (HR 12.9, 95%CI 1.8–94, p= 0.011, and HR 38.3, 95%CI 3.1–467, p= 0.004, respectively) as independent predictors for LR in their multivariate analysis (17).

Oh et al. (15), Li et al. (16), and Minervini et al. (18) addressed tumor grade in their studies involving PN but did not correlate it with surgical margin or LR.

Çinar et al. found that patients with LR after PN had PSM with low-grade tumors and NSM with high-grade tumors (19). However, in their RCT, Wu et al. did not report an association between high-grade tumors and LR, despite including 21 high-grade tumors patients (13.8%) (14).

In Radfar et al.’s study, there was no statistically significant difference in tumor grade between PSM and NSM groups (p=0.601) or between patients with and without LR (p=0.612) in ccRCC subtype. However, they did not specifically assess the association of PSM in high-grade tumors and its role in LR (20).

In a retrospective study, Shah et al. observed PSM as a risk factor for recurrence in high-risk tumors (pT2-3a or grades ≥III) but not in low-risk cases (pT1 or grades ≤II) (4).

Khalifeh et al. evaluated tumor size, growth pattern, pathological stage, tumor grade, multiple tumors, or surgeon learning curve in univariate and multivariate analysis but did not find any significant predictors for PSM (22).

### 3.6 Nephrometry score

Direct comparison between the RENAL score and LR was not reported in studies, and in most cases, nephrometric scores such ad PADUA or RENAL were not used to assess tumor complexity, despite suggestions that higher complexity could impact surgical outcomes. Morrone et al. reported a lower RENAL Score in the PSM group compared to the NSM group (6 vs. 7, p= 0.05) and identified it as a predictor of PSM in a multivariate analysis (p=0.001) (35). Conversely, a higher RENAL score was associated with NMS in multivariable analysis, potentially due to challenges in locating smaller intra-parenchymal masses, leading to surgeon overconfidence in technically easier procedures (35).

### 3.7 Perioperative outcomes

A longer warm ischemia time (WIT) was identified as a significant predictive factor for PSM, as expected. Morrone et al. found that PSM was associated with a longer WIT (20.4 ± 10.3 and 17.8 ± 8.5 minutes for PSM and NSM, respectively; p = 0.001). Additionally, they reported no significant association between PSM and complications during surgery (6.7% and 3.9% for PSM and NSM, respectively, p = 0.168), retroperitoneal approach (10.9% and 7.8% for PSM and NSM, respectively; p = 0.279, sample size:168), discordant approaches (41.2% and 42.8% for PSM and NSM, respectively, p = 0.845), or on-clamp procedure (91% and 86.8% for PSM and NSM, respectively; p = 0.198) (35).

### 3.8 Time-to-LR

Most LR after surgery occurs within the first 2 years, according to evidence in the literature, with median follow-up periods ranging from 24 to 162 months. Radfar et al. reported a mean time to LR of approximately 9 months (range 2–18 months) but suggested that LR occur later (20). Tellini et al. described a median time to LR of 43 months (IQR 17–68) for PSM and 56 months (IQR 26–96) for NSM (7).

### 3.9 Survival rates

There is no consensus regarding the statistical correlation between surgical margins and recurrence rates or specifical survival rates. Marchiñena et al. described significantly higher LR-free survival rates at 3-years post-PN in NSM patients compared to PSM patients (96.4% (95% CI 91.9–100) vs. 87.8% (95% CI 71.9–100), respectively; p= 0.02) (17).

Radfar et al. reported that the occurrence of LR in PSM patients did not affect OS rates compared to NSM patients (20).

Brassetti et al. introduced a novel trifecta for RAPN consisting of NSM, no major complications (CD≥3), and ≤30% postoperative eGFR reduction, aiming to enhance reproducibility and reliability.

From a multicenter, multinational database of non-metastatic cT1-2 RCC patients, they demonstrated that this trifecta could be a significant predictive factor for various outcomes including recurrence, mortality, and renal function deterioration (9).

Morrone et al. found no difference in RFS (p=0.68), MFS (p=0.71), or OS (p=0.88) between PSM and NSM groups in their study (35).

In the study of Khalifeh et al., the 3-year RFS (47% and 98.3%) and MFS rates (63% vs. 99.5%) were lower in PSM compared to NSM patients (22).

### 3.10 Management of LR

Managing LR after PN poses challenges and has been a controversial (20). Studies suggest considering active surveillance (AS) for PSM cases, as radical prostatectomy or re-resection of PSM may result in over-treatment (1, 3, 20). EAU guidelines recommend counselling PSM patients about the increased LR risk and the need for strict follow-up (1). For LR management, local treatment is recommended when possible and in the absence of significant comorbidities (1). Recurrent tumor growth in the regional LNs or ipsilateral adrenal gland might represent the metachronous spread of metastases. After the treatment for localized disease alone, systemic progression is common (2).

## 4 Discussion

The literature review has highlighted diverse approaches and perspectives regarding the association between PSM and LR. While some studies have reported PSM as an independent predictor of LR, others have found no significant correlation between the two variables. The variability in findings underscores the complexity of this relationship and suggests the need for further investigation to elucidate the underlying mechanisms. Management strategies for LR after PN remain subject of debate and controversy. AS is suggested for PSM cases to avoid over-treatment, as radical prostatectomy or re-resection of PSM may not necessarily improve outcomes and could potentially lead to unnecessary morbidity. Consistent with this approach, guidelines recommend counseling PSM patients about the increased risk of LR and emphasize the importance of strict follow-up. In cases where local treatment is warranted, surgical intervention for LR after PN should be considered judiciously, weighing the benefits against the potential risks and patient-specific factors. The decision to pursue surgical treatment should take into account the feasibility of complete resection, the presence of metastatic disease, and the overall clinical condition of the patient. Moreover, the association between local and systemic treatment modalities may offer adjunctive advantages in terms of survival, particularly in the presence of metastases.

The limitations of the current review, including retrospective study designs, heterogeneous methodologies, and varying follow-up periods, underscore the need for well-designed prospective studies with standardized protocols to elucidate the optimal management strategies for LR after PN. Additionally, future research should focus on identifying biomarkers or predictive models that can reliably stratify patients at risk of LR, facilitating personalized treatment approaches and improving clinical outcomes.

In conclusion, the management of LR after PN for RCC remains a complex and evolving field, characterized by diverse perspectives and approaches. While AS may be appropriate for select cases, careful consideration of surgical intervention is essential, taking into account individual patient factors and the potential benefits of adjunctive treatments. Further research is warranted to address the current gaps in knowledge and to refine clinical management strategies for optimizing outcomes in patients with LR after PN for RCC.

## Author contributions

LD: Conceptualization, Data curation, Writing – original draft. AC: Data curation, Visualization, Writing – review & editing. AA: Writing – review & editing. PC: Writing – review & editing. DD: Writing – review & editing. EM: Writing – review & editing. GL: Writing – review & editing. DV: Writing – review & editing. FS: Writing – review & editing. GB: Writing – review & editing. UF: Writing – review & editing. MM: Writing – review & editing. FM: Writing – review & editing. GG: Writing – review & editing. RF: Writing – review & editing. AP: Conceptualization, Data curation, Validation, Writing – original draft.
